# Feline Polycystic Kidney Disease: An Update

**DOI:** 10.3390/vetsci8110269

**Published:** 2021-11-08

**Authors:** Lorie Schirrer, Pablo Jesús Marín-García, Lola Llobat

**Affiliations:** Department of Animal Production and Health, Veterinary Public Health and Food Sciences and Technology (PASAPTA), Facultad de Veterinaria, Universidad Cardenal Herrera-CEU, CEU Universities, 46113 Valencia, Spain; schirrer.lorie@alumnos.uchceu.es

**Keywords:** cat, control disease, feline polycystic kidney disease, hereditary pathology

## Abstract

Polycystic kidney disease (PKD) is a disease that affects felines and other mammals, such as humans. The common name is autosomal dominant polycystic kidney disease (ADPKD) and causes a progressive development of fluid-filled cysts in the kidney and sometimes in other organs as the liver and pancreas. The formation and growth of cysts progress slowly, causing deterioration of kidney tissue and a gradual decrease in kidney function, leading to irreversible kidney failure. Feline PKD or ADPKD in humans are hereditary pathologies of autosomal dominant transmission. ADPKD is one of the genetic diseases with the highest prevalence in humans. In cats, this disease also has a high prevalence, mainly in the Persian breed, being one of the most common feline genetic diseases. Imaging tests seem to be the most reliable method for diagnosis of the disease, although more genetic tests are being developed to detect the presence of the responsible mutation. In this review, we summarize the current knowledge about feline PKD to guide future research related to an adequate diagnosis and early detection of causal mutations. It can allow the establishment of selection programs to reduce or eliminate this pathology in feline breeds.

## 1. Introduction

Polycystic kidney disease (PKD) is an inherited disease that causes a progressive development of fluid-filled cysts in the kidney and, sometimes, in other organs such as liver and pancreas [1]. Cyst formation and growth progress slowly, causing deterioration of kidney tissue and a gradual decrease in kidney function, leading to irreversible kidney failure. This hereditary pathology of autosomal dominant transmission represents one of the genetic diseases with the highest prevalence in humans, where it is called Autosomal Dominant Polycystic Kidney Disease (ADPKD). ADPKD affects from 1:200 to 1:1000 people [2]. In cats, this disease also has a high prevalence, mainly in the Persian breed, being in this breed one of the most prevalent feline genetic diseases, along with diabetes and feline lower urinary tract disease [3,4,5]. However, the Persian breed is not the only breed affected by this disease. Other breeds such as the Exotic Shorthair, Himalayan, British Shorthair, American Shorthair, Burmilla, Ragdoll, Maine Coon, Neva Masquerade and Chartreaux breeds can be affected by this pathology [3,6,7,8,9,10]. Currently, imaging tests such as ultrasound seem to be reliable methods in the diagnosis and monitoring of the disease [4,11,12]. Additionally, multiple genetic tests have been developed to determine the presence of the responsible mutation, giving breeders, owners and clinics the ability to easily detect PKD at an early stage [4,8,13]. Thus, these early diagnosis techniques would allow the establishment of selection programs to reduce or eliminate this pathology in cats.

In this review, we summarize the epidemiological and clinical findings of this disease, genetic aspects, and management of disease.

## 2. Epidemiology of Feline PKD

The first study that analyzes the prevalence was carried out in the United States, where the prevalence of PKD in Persian cats was around 38% [11]. Later, other authors studied the prevalence in this breed in Australia (50%), the United Kingdom (49.2%), France (40.45%), Italy (41%), Slovenia (36%), Taiwan (15.7%), Iran (36.38%), Japan (46%) and Brazil (5%) [6,12,13,14,15,16,17,18,19]. In these studies, no statistically significant differences were documented between males and females, suggesting that the inheritance of the disease is not sex-linked [14,20]. It has been studied less in other feline breeds such as Neva Masquerade cats or Siberian cats [9,21], although some authors suggest that it could be present in all feline breeds, since around 80% of all current feline breeds have had some type of cross with the Persian breed, so they could have inherited the mutation that provokes the disease [22]. In fact, Lyons et al. (2004) concluded that PKD affects 6% of the total feline population around the world. This would represent that this pathology is the most prevalent genetic disease in cats [5]. This high prevalence and the lethal nature of the disease justify the increasing interest of veterinarians and breeders in this disease. Thus, in 2001, the English organization Feline Advisory Bureau (FAB), now International Cat Care (ICC), developed a research program with selective purpose in the United Kingdom. The main aim was to identify affected cats, create a registry of animals with the genetic tests, and thus allow breeders to select healthy animals in their breeding lines [23]. More recently, a study in Brazil indicates a significant decrease in feline PKD prevalence (5% in Persian cats), which could be related to the first success of genetic counseling [19]. However, more studies should be carried out to evaluate the repeatability of the data obtained and eliminate possible mistakes. Although this is the first study to analyze the results of genetic counseling in reducing the prevalence of the disease, there is no doubt that good advice from veterinarians in the genetic selection of cats can greatly reduce this severe pathology.

## 3. Genetic Aspects of Feline PKD

**Figure 1:**
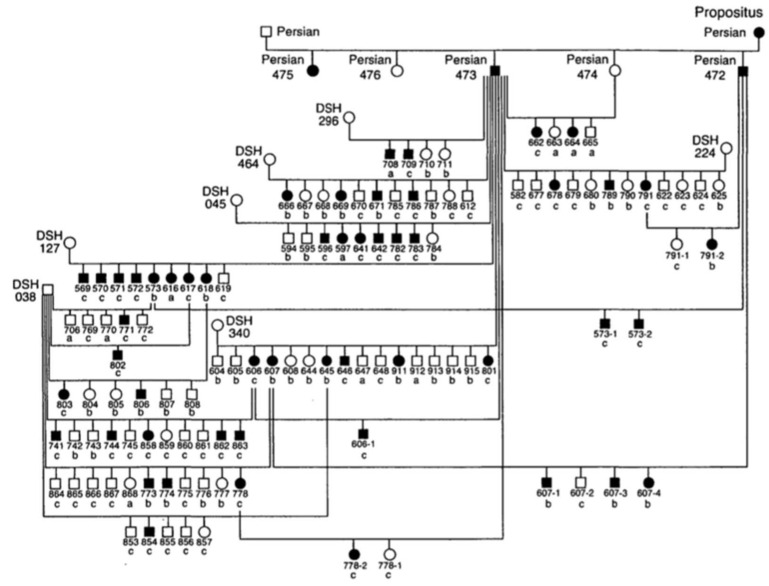
Pedigree of the colony of cats affected by Polycystic kidney disease (PKD). Square = male, circle = female, black symbol = affected cats, white symbol = unaffected cats, DSH = domestic shorthaired cat, Persian = Persian cat, a = renal histology, b = ultrasound, c = renal histology and ultrasound [25].

Since the 1970s, several cases of feline PKD have been described in the literature. In 1990, Biller et al. hypothesized for the first time a hereditary nature for the disease, after studying a 6-year-old female Persian cat that had been crossed with a healthy male Persian cat [24]. The female was referred for hematuria and polyuria-polydipsia, whose diagnosis of PKD was made by ultrasound and confirmed by anatomical-pathological examination. This female had given birth to five kittens divided into two litters. Ultrasound examination of four kittens showed that two males and one female were affected by PKD [24]. In 1996, the same authors identified the type of inherited transmission of PKD in a study carried out in a colony of cats. For this study, the authors created an experimental pedigree of affected cats from the case previously studied in 1990 (Figure 1). To create the colony, two litters obtained by crossing this female with a healthy Persian male were used. All affected cats in this family were identified by ultrasounds by anatomical-pathological analysis, or both [25].

The results of this study made it possible to identify the autosomal dominant mode of transmission of PKD. The statistical analysis showed proportions that corresponded to an autosomal dominant inheritance with complete penetrance. The animals suffered from the disease only with the presence of a defective allele in their genotype and with a penetrance of 100% (it occurred in all individuals) [25]. To date, no homozygous animals have been found for the mutation of *PKD1* gene, which reinforces the idea that it is lethal in utero [5,8,17].

Feline PKD was first identified in 1969 in some sporadic cases [26], and it was described in more detail in the 1990s, with the identification of the hereditary transmission and its similarity to humans ADPKD [25]. The following studies made it possible to discover the implicated gene called *PKD1* and the mutation that provokes the disease [5,27]. The *PKD1* and *PKD2* genes code for the polycystin-1 and 2 proteins. Mutations in these genes are responsible of 85% and 15% of human ADPKD cases, respectively, and affect the kidney and the bile duct [28]. The presence of cysts depends on animal age, so 90% of presumed gene carriers will have cysts that can be identified based on the age of the animal [2,29]. For example, a clinical case was described in six kittens that died at seven weeks of age, in which the necropsy revealed the presence of renal and bile duct cysts, undiagnosed by the young age of the animals [30]. Recent studies in human medicine reveal high genetic and clinical heterogeneity of this disease [31]. Thus, although now only the *PKD1* gene has been identified as the cause of the disease in cats, several studies found cases of animals with kidney cysts and a wild-type genotype of the *PKD1* gene. This raises the existence of other responsible mutations and, therefore, a possible genetic heterogeneity of feline PKD [3,8,32].

### 3.1. Gene and Mutation Involved in PKD and Their Identification

In 2005, Young et al. conducted a study to identify the gene involved in feline PKD [27]. To search the molecular markers, which are used to establish genetic maps and locate the position of genes in the genome, the authors were inspired by the disease present in humans and mice. Thus, mutations in the *PKD1* and *PKD2* genes are responsible for ADPKD in humans, mutations in the *PKDH1* (Polycystic kidney and hepatic disease 1) gene are responsible for ARPKD in humans, and mutations in *Nek8* (NimA-related kinase 8) gene cause PKD in mice and zebrafish [32,33,34,35]. Forty-three microsatellites were chosen from the feline genetic maps, based on known homologies to human chromosomal regions containing the *PKD1*, *PKD2*, *PKDH1*, and *Nek8* genes. Linkage analysis, using seven pedigrees of Persian cats segregating for PKD showed a significant and non-recombinant link between the PKD disease locus and the FCA476 marker located on chromosome E3. These data suggested that the *PKD1* gene or another gene within this region could cause feline PKD [27]. The authors explained that since the Persian breed is relatively inbred, PKD is unlikely to be genetically heterogeneous, thus they suggested that the *PKD1* gene should be investigated for a causal mutation.

**Figure 2:**
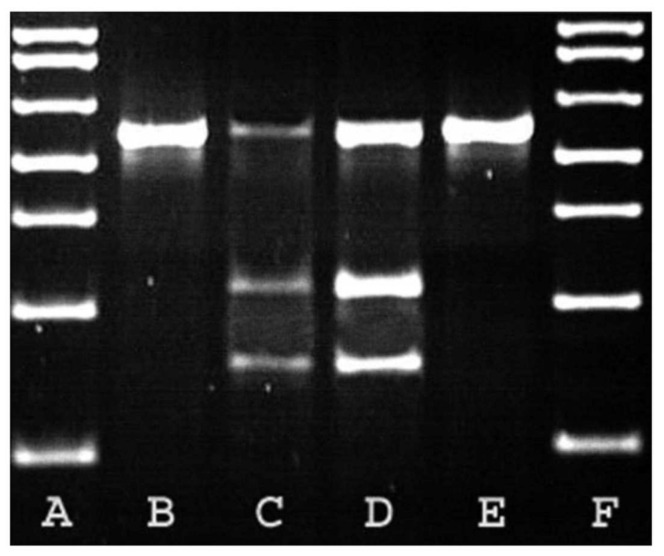
Restriction Fragment Length Polymorphism (RFLP) analysis for feline Polycystic Kidney Disease (*PKD1*) mutation. B and E = unaffected cats, C and D = affected cats, A and F = molecular weight markers [5].

Lyons et al. sequenced the feline *PKD1* gene to determine the causal mutation of feline PKD, with Polymerase Chain reaction (PCR) amplification and a study of its products by the Rapid Fragment Length polymorphism (RFLP) method [5]. The authors identified a nucleotide variation, characterized by a substitution of a pyrimidine base (cytosine, C) for a purine base (adenine, A) at position 3284 of exon 29 of the feline *PKD1* gene (c.10063C > A), which is present at a prevalence of 30% in the Persian breed [36]. This mutation results in the premature appearance of a stop codon in the messenger RNA, which causes the loss of 25% of the C-terminal in the formation of the polycystin-1 protein, generating a mutated protein. This change in a single base also gives rise to a single restriction point in the amplification products of exon 29, which allowed confirmation of the presence of the mutation in all cats in the study by RFLP analysis. The restriction enzyme MLY1 produces the digestion of exon 29 of 559 bp into two fragments of 316 and 243 pb in affected cats. All affected cats had the 559 pb wild-type fragment and the two digested fragments, while the unaffected cats had only the 559 pb wild-type fragment (Figure 2). This mutation presents a high prevalence in some feline breeds as Persian (46%), Scottish Fold (54%) and American Shorthair (47%) cats [18].

### 3.2. New Perspectives in Genetic Cause of Feline PKD

There is a hypothesis of the existence of other mutations responsible for PKD in cats. Some animals with renal cysts, visualized by ultrasound or histopathological examination, were homozygous in the wild, that is, without the presence of the mutation c.10063C > A. In fact, several studies found a small percentage (around 5%) of homozygous wild animals in their results. These data suggest that other mutations could cause feline PKD and even that environmental factors, such as anxiety or epigenetic factors, could have an influence in the onset of the disease [8,13,33,37]. However, it must be considered that cats with renal cysts and wild homozygous ones could present other pathologies different from PKD and not another genetic form of it, so it is important to carry out a differential diagnosis. In this differential diagnosis of feline PKD, other conditions such as simple renal cyst, cystic disease as a consequence of chronic renal failure or a cyst caused by a tumor must be considered [5]. A recent study indicated that there is a wide range of progression and severity in the disease, which also suggests the involvement of other factors that modify the expression [36]. In fact, Guerra et al. presented the case of a 1-year-old male Persian cat with PKD associated with congenital liver fibrosis, and in which it was shown by genome sequencing that it did not present the C3284>A in the exon 29 of the *PKD1* gene [38]. The authors suggested that other genes such as *PKDH1* (characteristic of the recessive form ARPKD in humans), not yet described in cats, could be involved in the pathogenesis of this phenotype. In humans, PKD exhibits genetic heterogeneity, with autosomal dominant and autosomal recessive inheritance patterns in several genes. Thus, mutations in polycystin-1 (*PKD1*), polycystin-2 (*PKD2*), neutral alpha-glucosidase AB (*GANAB*, or *PKD3*), and the homologous subfamily DnaJB11 (*DNAJB11* or *PKD6*) genes, are associated with ADPKD disease. In addition, fibrocystin (encoded by the *PKDH1* and *PKDH4* genes) and the DZIP1L protein (encoded by the *DZIP1L* and *PKDH5* genes) are associated with ARPKD disease [37,39]. In fact, more than 1270 mutations causing kidney disease have been determined in the *PKD1* gene in humans, although a single causal mutation has been described in cats [5,40]. These considerations justified the study by Bilgen et al. (2020), where the authors analyzed a family of Siamese cats with a history of hereditary kidney disease [41]. It was carried out using the whole genome sequencing (WGS) method, which allows a complete sequencing of the genome. The study revealed several new variations in all genes, as well as missense mutations, and point mutations in the *PKD2*, *DZIP1L*, and *PKDH1* genes. Recently, a study carried out by whole exome sequencing (WES) has explored a new variant, specifically a novel frameshift mutation in polycystin 2 (PKD231) in a Siberian cat, as a possible cause of feline PKD [21]. These several mutations affect ciliary structures and can result in different severities of the disease, suggesting a possible epistatic interaction. These mutations should be studied, and a combined use of clinical, histopathology, and WGS and WES tests is recommended to cover all candidate genes.

## 4. Pathophysiology and Clinical Features

Regarding the pathogenesis of the disease, there are still many causes under study, and the pathogenesis processes are not well understood. In humans, abnormalities in gene expression, cell polarity, fluid secretion, and apoptosis have been hypothesized. It seems that the formation of the cysts could be related to a hyperplasia of the tubular epithelium, which causes a partial obstruction of the tubules, preventing the flow of urine [7]. The mutation of the *PKD1* gene triggers the modification of the polycystin-1 protein, which is expressed in the primary cilium, a flagellar structure originating from the tubular cell and in contact with the urinary flow. These cilia are organelles that function in fluid transport and chemo and mechanoreceptors [42,43]. Currently, the precise function of polycystin-1 is unknown, but it appears to be involved in cell–cell and matrix–cell interactions [44]. The predominant hypothesis about the pathogenesis of ADPKD focuses on the role of the cilium–centrosome complex of tubular epithelial cells. Disorders that result in defects of this complex are called “ciliopathies” and many of the associated disorders have renal cysts as a part of their pathology [40,44]. The cyst formation process seems to occur through the combination of increased cell proliferation, fluid secretion, and extracellular matrix alterations, so the loss of polarization of the cilia would alter the water reabsorption function, developing cysts in the parenchyma [45].

Feline PKD is characterized by the presence of cysts, in variable number and size, in the renal parenchyma. The cysts are present from birth, they form in the cells of the renal tubules and most of them are observed in the cortex or in the cortico-medullary area [18]. These cysts increase in number and size proportionally with age, which explains that many cats are still subclinical for several years [36]. The clinical signs of PKD are not pathognomonic for this condition, as it manifests as chronic renal failure. The average age of appearance of clinical signs is established at seven years, but they can appear between three and ten years [4,17,26]. In general, the clinical signs observed on the basis of history can be apathy, anorexia, weight loss, bad appearance of the coat, polyuria and polydipsia, as well as gastrointestinal disorders [26,46,47]. On clinical examination, general dehydration, pale mucous membranes can be observed, as well as increased volume and irregular contour of the kidneys on palpation. Although curative treatment does not exist, these clinical signs can be alleviated with palliative treatment.

In affected cats, laboratory findings are not specific, mainly indicating renal failure (azotemia, hyperphosphatemia, non-regenerative anemia, and proteinuria). However, clinical stages can be highly variable, as demonstrated in a recent study where several young animals presented azotemia with a remarkably high creatinine concentration, compared to older animals with less important values [18]. Several authors have found that there is significant individual variation in the progression of disease, although there is still no conclusive evidence [36]. The variability observed between cats, as well as the variability that can occur between the two kidneys of the same cat, suggests that other factors can change the expression and progression of the disease. Clinically relevant aspects include renal manifestations, but there are also extrarenal manifestations where liver involvement is the most common. The hepatic cyst is an extrarenal manifestation that occurs in some cases of feline PKD [38]. In humans and cats, the rate of matching liver and kidney cysts is approximately 80% and 12.6%, respectively [36,48]. However, in humans there is also a marked dilatation of the bile ducts associated with cysts, while cats do not show other hepatobiliary lesions, so liver cysts could have a different pathogenesis from humans ADPKD [38]. Furthermore, there were no statistically significant differences between the age of the cat and the stage of disease with the presence of liver cysts. The stage of the disease could not be related to the formation of liver cysts, although some studies reported cases of related liver fibrosis in cats with PKD [36,38]. Nowadays, clinical signs associated with liver failure have rarely been found, and the inherited nature of this process has not been established.

In cats, mutated polycystin-1 seems to play a significant role in cell proliferation and differentiation of the tubular epithelium, in addition to known antiapoptotic activity [49]. Thus, the balance between tubular degeneration, activation of necrosis and apoptosis is a key factor in the appearance of cysts. In this way, the induction of cell death in affected cells could be related to the pathogenesis of the disease [41]. In addition to cystic structures, fibrosis of kidney tissue and increased expression of transforming growth factor beta (TGF-β) around these fibrous areas were observed, suggesting that in animals with PKD, renal failure may also be caused not only by cyst formation but renal fibrosis could be a crucial factor [41,49]. Another factor that must be considered is that there are other mutations that cause ciliopathies, as well as other diseases that can generate kidney cysts [50]. These pathologies can mimic PKD and should be considered as phenocopies when studying the mechanisms of PKD [46]. To date, the etiopathology of the disease is not defined and is based on different hypotheses. It is being studied in both humans and veterinary medicine to explore the differential formation of kidney cysts.

## 5. Diagnosis of Feline PKD

Diagnosis of PKD cannot be established by the only clinical features. For example, renal palpation can reveal nephromegaly, but this can be caused by other pathologies. The previously mentioned clinical signs, the evidence of renal failure by laboratory findings and epidemiological data (mainly, feline breed) can guide the diagnosis of disease [14]. However, current methods of choice are imaging tests, mainly ultrasound, and recently developed genetic study methods.

### 5.1. Imaging Diagnostic

The use of imaging tests is essential in the feline PKD diagnosis. Radiography and intravenous urography can be used in more advanced cases, when there are multiple, large cysts. However, the examination with the most success is that of ultrasound, which allows a quick and reliable diagnosis to be obtained, and it is the only current method that decides the severity and progression of the disease [36]. In addition, ultrasound is widely available and non-invasive, safe, cheap and effective in detecting the presence of kidney cysts [47]. The cysts are seen as hypo- to anechoic spherical cavities, which may be associated with a later contrast, with a variable size from one to more than twenty millimeters. For an improved diagnosis, renal ultrasounds should be completed with a liver ultrasound to assess the presence of other cysts [13]. The sensibility, specificity and repeatability of ultrasound are around 91–96.2%, 91–100% and 100%, respectively [12,20,33]. The clinical recommendation is that the ultrasound be carried out by a specialist veterinarian and by a high-resolution ultrasound machine with a 7.5–12 MHz multifrequency linear transducer [11,12,17,51]. When performing imaging diagnosis, it must be considered that cysts may be more difficult to detect in the medulla than in the cortex, due to the echogenicity of the medulla, and that the hypoechoic nature of the medulla can lead to false positives [14,20]. Yu et al. (2019) compared the different imaging methods in the diagnosis and follow-up of PKD (CT or Computerized Axial Tomography, MRI or Magnetic Resonance Imaging, and ultrasound) [36]. In humans, CT or MRI methods are often used in the diagnosis of ADPKD, and it has been shown that measurements of renal volume are the best biomarker to check and predict disease progression. In summary, while MRI is preferable to CT in human medicine due to radiation exposure, CT is more practical to assess disease progression for feline PKD, due to the rapid acquisition of images that only requires sedation or light anesthesia, lower cost, and greater availability. In addition, estimates of Total Kidney Volume (TKV), Total Cyst Volume (TCV) and Fractional Cyst Volume (FCV) were found to be valid in cats to assess disease stage. These results appear very promising for the future and could lead to a better follow-up of the disease, as well as a risk classification for renal failure, such as to those of humans [36]. In Persian cats, the diagnosis will be carried out from 10 months of age preferably, since it is difficult to detect the cysts in young cats [6,12,21]. In 2018, a diagnosis criterion with ultrasounds and genetic analysis of the *PKD1* gene was carried out, and the results were that the diagnosis depended on the age of the animal (Table 1) [47].

Given the difficulty and uncertainty of diagnostic imaging, molecular diagnostics have been developed.

### 5.2. Genetic and Molecular Diagnosis

Based on the identification of the gene involved in the development of PKD [5], several methods have been established for the identification of the mutation responsible for the disease. The PCR method is mainly used to identify and amplify the DNA fragment of interest. There are different variants of PCR that have been used and confirmed in different studies.

For example, Lyons et al. (2004) developed an RFLP-PCR test for the identification of the gene involved [5]. Later, Helps et al. (2007) used the method of real-time PCR or quantitative PCR, which was reliable and faster than the earlier technique [8]. In 2010, Lee et al. developed a new method of ARMS-PCR (Amplification-refractory mutation system-Polymerase Chain Reaction), which resulted in 100% sensitivity and specificity. This method presents many advantages in terms of time to obtain the result, the low quantity sample size needed and its low cost [13]. In 2014, Scalon et al. tested the TD-PCR (Touchdown-Polymerase Chain Reaction) method, adapted from the multiplex PCR of Lee et al. and confirmed that these techniques can produce the same results as the complex and expensive RFLP-PCR technique [22]. In general, these techniques are different alternatives but present a perfect agreement in terms of the diagnostic value of PKD. Today, the most widely used techniques at the practical level are real-time PCR and RFLP-PCR [10].

From the perspective of elimination of PKD within the Persian breed and similar breeds, it is necessary to diagnose affected cats as soon as possible to allow breeders to program their mating schedules. Thus, these studies that describe the various molecular methods seem to show a good correlation between the results of genetic tests and those obtained from the ultrasound examination, although with the advantage for molecular methods of an early application [4,51]. The fact that the pathology is due to a point mutation makes molecular tests an adequate tool for the diagnosis of PKD, but their limited access and excessive cost can restrict their application to the daily clinic, for which ultrasound has shown to be the most profitable imaging modality for renal phenotypic evaluation in potentially affected animals [47].

Thus, genetic testing is the method of choice to confirm the presence of the causal mutation and make an early diagnosis, especially in animals younger than 4 months, and ultrasound is the method of choice to diagnose polycystic kidney disease and to monitor the progression of the disease. In conclusion, several authors agree on the recommendation of the synergistic use of both tests to reach a complete medical diagnosis, especially in breeding cats, to plan detection programs for feline PKD [4,33,51].

## 6. Conclusions

Feline polycystic kidney disease is a disease with high prevalence in some feline breeds such as the Persian breed. This disease is characterized by chronic renal failure, appears in animals between three and ten years of age and leads to severe and irreversible kidney failure. Current treatment is only palliative. Diagnosis of imaging methods is feasible, but it must be complemented with genetic and molecular tests to avoid confusing the diagnosis with other ciliopathies. Currently, genetic diagnosis is made by detecting the mutation c.10063C > A in *PKD1* gene, but recent studies with WGS and WES methodology have indicated that there are probably other genetic variants associated with this disease, both in this same gene, as in others such as *PKD2*, *DZIP1L*, and *PKDH1*. Further studies to determine the mutations associated with the onset of the disease are necessary, not only for an early diagnosis but also to apply this knowledge to the selection programs of some feline breeds with high prevalence of this disease.

## Figures and Tables

**Table 1:** Diagnosis criteria of feline PKD according to the age of the cat.

Age (Months)	Diagnostic Criteria for Kidney Ultrasound
≤15	≥1 cyst
16–32	≥2 cysts
33–49	≥3 cysts
50–66	≥4 cysts

Data extracted from Guerra et al. (2018) [47].

## Data Availability

Not applicable.
